# Robust genetic machine learning ensemble model for intrusion detection in network traffic

**DOI:** 10.1038/s41598-023-43816-1

**Published:** 2023-10-11

**Authors:** Muhammad Ali Akhtar, Syed Muhammad Owais Qadri, Maria Andleeb Siddiqui, Syed Muhammad Nabeel Mustafa, Saba Javaid, Syed Abbas Ali

**Affiliations:** 1 Department of Computer and Information System Engineering, NED University of Engineering and Technology, Karachi, Pakistan; 2 Department of Computer Science and Information Technology, NED University of Engineering and Technology, Karachi, 75270 Pakistan; 3 Department of Physics, NED University of Engineering and Technology, Karachi, Pakistan

**Keywords:** Information technology, Scientific data, Engineering, Mathematics and computing

## Abstract

Network security has developed as a critical research subject as a result of the Rapid advancements in the development of Internet and communication technologies over the previous decades. The expansion of networks and data has caused cyber-attacks on the systems, making it difficult for network security to detect breaches effectively. Current Intrusion Detection Systems (IDS) have several flaws, including their inability to prevent attacks on their own, the requirement for a professional engineer to administer them, and the occurrence of false alerts. As a result, a plethora of new attacks are being created, making it harder for network security to properly detect breaches. Despite the best efforts, IDS continues to struggle with increasing detection accuracy while lowering false alarm rates and detecting new intrusions. Therefore, network intrusion detection enhancement by preprocessing and generation of highly reliable algorithms is the main focus nowadays. Machine learning (ML) based IDS systems have recently been implemented as viable solutions for quickly detecting intrusions across the network. In this study, we use a combined data analysis technique with four Robust Machine learning ensemble algorithms, including the Voting Classifier, Bagging Classifier, Gradient Boosting Classifier, and Random Forest-based Bagging algorithm along with the proposed Robust genetic ensemble classifier. For each algorithm, a model is created and tested using a Network Dataset. To assess the performance of both algorithms in terms of their ability to anticipate the anomaly occurrence, graphs of performance rates have been evaluated. The suggested algorithm outperformed other methods as it shows the lowest values of mean square error (MSE) and mean absolute error (MAE). The experiments were conducted on the Network traffic dataset available on Kaggle, on the Python platform, which has limited samples. The proposed method can be applied in the future with more machine learning ensemble classifiers and deep learning techniques.

## Introduction

The continuous evolution of internet and communication technologies has triggered an extensive transformation, amplifying the scale and data capacity of modern networks. This progression, while empowering digital connectivity, has also introduced a proliferation of novel cyber threats, amplifying the intricacies involved in detecting breaches within network security. Furthermore, the persistent specter of intruders seeking to orchestrate diverse attacks within networks accentuates the gravity of these challenges^1,2^.

A combination of technologies known as cyber security safeguards computers, networks, and data against hackers. It may contain firewalls, antivirus software, and intrusion detection systems (IDSs). Unauthorized traffic, unauthorized logins, data duplications, data destructions, and abnormal behaviors are all examples of intrusions that an IDS can detect. For years, many intrusion detection systems have been utilized. Signature-based misuse detection based on known behavior and Anomaly-based detection based on anomalous behavior are the two main types of IDS mentioned. Current IDS have several flaws, including their inability to prevent attacks on their own, the requirement for a professional engineer to administer them, and the occurrence of false alerts. Data mining techniques are being used in this industry to alleviate some of these issues. However, in most circumstances, high detection comes at the expense of increased computing time. Nowadays, it is critical to detect threats quickly and automatically with as few false alarms as possible^3^.

Network security has developed as a critical research subject as a result of the current interest and advancement in the development of internet and communication technologies over the previous decade. To protect the security of the network and all its related assets within cyberspace, it uses tools such as firewalls, antivirus software, and intrusion detection systems (IDS)^4^. A network-based intrusion detection system (NIDS) is an attack detection technique that delivers the needed security by continuously monitoring network traffic for malicious and suspicious behavior. Designing intrusion detection systems (IDS) is significantly hampered by the emergence of malicious software (malware). The main issue in identifying unknown and obfuscated malware is that the creators of the infection utilize various evasion tactics for information concealment to evade detection by an IDS^5^. Malicious attacks have become more complex. Additionally, there have been more security risks like zero-day attacks that are aimed at internet users. Consequently, since the usage of information technology has permeated our daily lives, computer security has become crucial^6^. There have been high-profile cyber security attacks worldwide. So, it is necessary to create an effective IDS to find new, advanced malware. An IDS's goal is to quickly identify various malware types because a standard firewall is unable to do so. The creation of better IDSs has become crucial due to the rising prevalence of computer malware^7^.

In this research, we implemented four Machine learning algorithms, including the Naïve Baye, Decision Tree, K nearest Neighbors, and Bagging Classifier, as well as our own proposed Robust genetic ensemble classifier. A Network Dataset is used to generate and test models for each method. To achieve greater predictive performance, instead of using a single machine learning classifier, an ensemble approach is used which is a combination of many classifiers. The ensemble model's core tenet is that a collection of weak learners can be combined to create a strong learner, boosting the model's accuracy. Noise, variation, and bias are the key reasons why actual and predicted values differ when we apply any machine learning technique to predict the target variable. Ensemble aids in minimizing these factors (except noise, which is an irreducible error). Therefore, in this research, a combination of a data analysis technique with four Robust genetic ensemble learning algorithms, including the Voting Classifier, Bagging Classifier, Gradient Boosting Classifier, and Random Forest based Bagging algorithm, as well as the proposed genetic Robust genetic ensemble classifier are used^8,9^. A Network Dataset is used to generate and test models for each method. The word “Robust” depicts the data on which the model is based is robust in the presence of noise and outliers. Robust genetic ensemble learning algorithms are based on combinations of random forest-based bagging classifier, knn, decision tree, logical regression-based voting classifier, gradient boosting classifier, and random forest-based bagging classifier with evolutionary Genetic optimizer along with the cross-voting method. We used graphs and performance rates to analyze the performance of both algorithms in terms of their capacity to predict the occurrence of anomalies. Despite the collective endeavors of researchers, IDS confronts a persistent quandary: the simultaneous enhancement of detection accuracy while mitigating false alarm rates and adapting to emerging intrusion patterns. The application of machine learning (ML) techniques, particularly in the context of IDS, has garnered notable attention as a promising avenue for rapid intrusion identification across networks^10^. In parallel, the interdisciplinary realm of cybersecurity embraces an arsenal of defensive tools, encompassing firewalls, antivirus software, and IDS^11^. Notwithstanding these advancements, contemporary IDS systems exhibit certain limitations, notably their autonomy in thwarting attacks, the need for skilled engineering oversight, and their susceptibility to generating false alerts. To surmount these challenges, the integration of data mining techniques has emerged as a compelling approach, albeit often accompanied by augmented computational demands^12,13^. Beyond its theoretical significance, the outcomes of this study bear tangible implications for real-world cybersecurity practices. The adoption of robust machine learning algorithms, coupled with the innovative genetic robust ensemble classifier, holds the potential to fortify IDS systems against an evolving threat landscape. By equipping these systems with heightened predictive capabilities, organizations can proactively identify and neutralize threats, thereby curtailing potential damage.

### Contributions

1. Extensive feature selection and pre-processing of dataset to achieve better performance.
2. Introduction of a novel approach that integrates robust machine learning algorithms and a genetically robust ensemble classifier to enhance intrusion detection accuracy.

The rest of the paper is settled as “Related work” section, “Methodology” section, “Result and discussions” section and “Conclusion” section.

## Related work

The Systems for detecting intrusions may be based on known attacks (misuse detection based on signatures) or unusual behavior (intrusion detection based on abnormal behavior) (Anomaly-based detection). These two kinds of IDSs are created utilizing data mining methods. The core vector machine (CVM) is determined to be the superior intrusion detection classifier in^14^ when Robust Genetic Ensemble classifiers are examined. The Principal Component Analysis method is used in this feature selection process.

This method's drawback is that it can't be utilized on a single record, which means real-time systems can't use it. The many data mining classification techniques used in intrusion detection are covered in^15^. The results of this study make it clear that the type of assault will have an impact on how well classifiers work. Additionally, it discusses the many openly accessible data sets that can be utilized to develop and evaluate classifier models^16^. Suggest a CVM-based hierarchical method. It performs better than other classifiers for assaults like R2L and U2R.

A bagging robust genetic ensemble of decision trees is used by^17^ to identify network intrusions. Although it takes longer to train and test than CVM, it has an accuracy range of 81–99 percent. SVM is used in^12^ for intrusion detection. It offers a hybrid approach of filter and wrapper models for selecting key attributes^18^. Demonstrates how employing AdaBoost lowers the likelihood of false positives while increasing the detection rate of a Naive Bayesian network.

The concepts and characteristics of Core Vector Machines are covered in^19,20^. An overview of intrusion detection systems and the application of data mining in this field is given in the paper^21^. The ensemble classification and other classifications as a machine learning technique applied to image processing pattern recognition and NIDS have been investigated by several authors and are summarized here. The main aspect of network security is intrusion detection (ID). It is difficult to design and implement an intrusion detection system (IDS) that will effectively detect abnormal behaviors and attempts brought on by intruders in the network and computer system. Pairwise clustering and central clustering are the two broad categories into which clustering techniques can be subdivided. Using a data-pairwise proximity metric, the former method, also known as similarity-based clustering, groups comparable data instances together^22^.

The study and creation of algorithms that can generalize (i.e., learn) from small quantities of data are topics covered by machine learning approaches. Instead, then only following explicitly written instructions, these algorithms operate by creating models based on input and using those models to generate predictions or judgments. They are the best choices for intrusion detection jobs because they possess these qualities^23^.

Alternately, it may be claimed that systems built on machine learning are capable of changing their execution plans in response to fresh input. They are the best choices for intrusion detection tasks since they possess these qualities. Intrusion detection has effectively used machine learning. Several important machine learning techniques are as follows:

Artificial Neural Networks (ANNs) are used by some IDS designers, the NN learns to anticipate the actions of the different system users and daemons. NN can solve many of the issues encountered by rule-based systems if correctly developed and applied. The fundamental benefit of NN is their capacity to infer solutions from data without prior knowledge of the regularities in the data and their tolerance for imperfect data and ambiguous information. This method would need to be applied to ID, and data representing attacks and non-attacks would need to be fed into the NN for it to automatically modify its coefficients during training. To associate outputs—each of which represents a category of computer network connections, such as normal and attack—with matching input patterns, neural network parameters are improved during training (every input pattern is represented by a feature vector extracted from the characteristics of the network connection record). The neural network recognizes the input pattern when it is being utilized and tries to output the correct class. Long runtimes during learning are a common problem for ANNs caused by local minima^24^. The most complex ANN variants need even more processing power; hence they are frequently implemented on graphics processing units^25,26^.

The Machine learning techniques called classification and regression trees (CART) are used to build prediction models from data. These models are created by fitting a prediction model into each partition of the data after recursively partitioning the data. Consequently, the partitioning can be graphically depicted as a decision tree. The prediction error is assessed in terms of misclassification cost, and classification trees are designed for variables that are dependent and take a finite number of unordered values. Regression trees are used when the dependent variable has ordered discrete or continuous values, and the prediction error is commonly expressed as the square root of the difference between the observed and predicted values. The baseline will define what is “normal” for that person and will raise an alarm when abnormal behavior is seen or when the baseline is noticeably different. The greater false positive rate^27,28^ is the main problem.

The Machine that supports vectors Support Vector Machine (SVM) is a technique for supervised machine learning. Both classification and regression analysis can be done with it. The value of each feature is represented by the value of a specific coordinate, and this technique depicts each data point as a point in n-dimensional space (where n is the number of features available). After that, classification is carried out by locating the hyper-plane that clearly distinguishes the two groups. The main benefit of Support Vector Machines is that, because SVM is independent of feature space, it is less prone to overfitting the feature input from the input items. In this case, SVM's classification accuracy is pretty amazing or high. Using a set of accessible training data and the idea of information entropy, SVM creates decision trees quickly and accurately both during training and during testing. The algorithm chooses the property of the data from each node of the tree that divides its set of samples into subsets that are enriched in one class or the other. The normalized information gain serves as the splitting criterion. The attribute used to determine the decision is the one with the largest normalized information gain. Then, on the smaller sub-lists, the C4.5 algorithm repeats^29^.

Similar to the K-means technique, K-Medoids use a partitioning algorithm to create clusters. Instead of using the mean value of the objects in K-Means clustering, the centroid is the instance that is located closest to the center of the cluster. The term “reference point” refers to this centralized object. It reduces the separation between the centroid and the data points, hence reducing the squared error. When there are more data points, the K-Medoids algorithm performs better than the K-means algorithm. Because medoid is less influenced by outliers, it is robust in the presence of noise and outliers, but processing is more expensive^5,30,31^.

The K-nearest neighbor (KNN) is one of the simplest classification methods is this one. It determines the separation between various data points on the input vectors and places the unlabeled data point in the class of its closest neighbor. K is a crucial variable. The data point is given the class of its closest neighbor if k is equal to 1. When K is high, the prediction process takes a long time and influences accuracy by reducing the impact of noise^32^. The k-Means algorithm divides the input data into n instances and separates them into k distinct clusters, where k is a predetermined value. The closest cluster is given to each instance. For instance, in an assignment, every data point would be assigned to a cluster based on the minimal distance between the cluster's centroid and each instance. When applied to a small dataset, the K-Means technique runs faster. Execution time grows as the number of data points rises.

A network intrusion detection method based on evolutionary algorithm-optimized XGBoost is proposed to increase the speed and accuracy of model intrusion detection in a complex network environment. The XGBoost model is trained using the ten-fold cross validation method on the NSL-KDD data set, and the genetic algorithm is used to optimize the model parameters to predict and categorize whether the network is attacked. It not only resolves the issue of time-consuming and inefficient conventional grid search but also avoids the issue of low classification accuracy of basic machine learning models^33,34^.

A model of an extreme learning machine based on the optimization of the quantum beetle swarm method is put out in response to the low accuracy of intrusion detection. To combine the benefits of particle swarm optimization and beetle antennae search, this research first develops a quantum beetle swarm optimization algorithm. The beetle can move intentionally and instructively thanks to this ability to learn from both personal and communal experience, which also helps the algorithm's convergence performance. High-dimensional data problems are more challenging to solve in extreme learning machines. The least squares QR technique is used in this research to deconstruct the matrix, which can lower the computational complexity of the enhanced extreme learning machine.

The author in^35^ put out a unique ensemble SVM-based CGO algorithm to improve intrusion detection's classification accuracy in a huge data setting. Preprocessing is done on the dataset to get rid of redundant, noisy, and undesired data. The preprocessed data are now classified using ensemble SVM, and the CGO method is used to increase classification accuracy. The suggested solution makes use of a Hadoop infrastructure to quickly process the massive data instances.

Detailed literature review is summarized in Table 1.

**Table 1:** Summary of literature review.

Article	Year	Machine learning algorithm	Advantages	Disadvantages
Intrusion Detection System Using Feature Extraction with Machine Learning Algorithms in IoT^36^	2023	random forest, K-nearest neighbors, SVM	Better feature extraction for high accuracy	Need resources and computing time to achieve results and to understand what each image means
A machine learning-based intrusion detection for detecting Internet of Things network attacks^37^	2022	XGBoost, CatBoost, KNN, SVM, QDA, and NB classifiers	Enhanced preprocessing and data sampling	Lack real-world features
Designing a Network Intrusion Detection System Based on Machine Learning for Software Defined Networks^38^	2021	Decision Tree, Random, Forest, and XGBoost	Advanced preprocessing techniques	Fewer features used in the predictions of attacks
An End-to-End Framework for Machine Learning-Based Network Intrusion Detection System^39^	2021	KNN, random Forest, Naïve Bayes, Logistic Regression, Support Vector Machine, XGBoost, Decision Tree	Enhanced preprocessing	Lacks real world features
Intrusion Detection of Imbalanced Network Traffic Based on Machine Learning and Deep Learning^40^	2020	Random Forest (RF), Support Vector Machine (SVM), XGBoost, Long and Short-term Memory (LSTM)	Determination of samples to be expanded in the imbalanced network traffic in improve results	Low results

## Methodology

**Figure 1:**
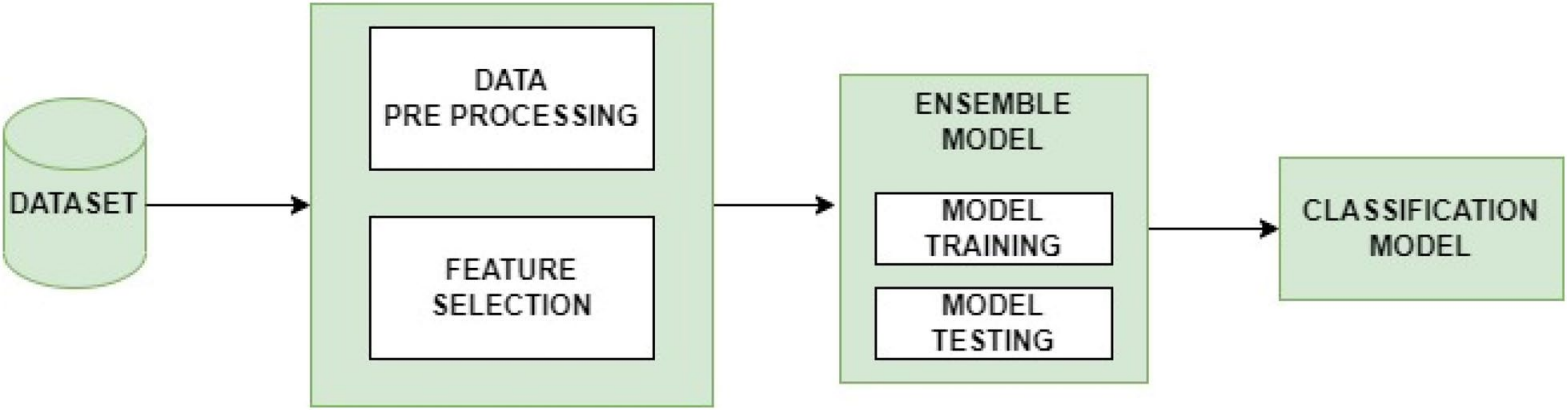
System diagram of proposed methodology.

In this work, we have chosen a robust genetic ensemble classifier random forest model-based bagging model using a genetic algorithm for optimization and Naive Baye, K nearest neighbor, decision trees, logistic regression, and bagging as its data analysis strategy. For each approach, a model is created and tested using network traffic dataset. We compared the findings of both algorithms using graphs and performance rates in order to assess how well they performed in terms of forecasting network incursion. Several procedures are carried out before experiments, including data preparation, data exploration, and model construction. These procedures are crucial for getting data ready so that it may be fed into models. Data transformation involves aggregating data, extrapolating data, creating dummies, and reducing the number of variables. Combining data involves combining or joining datasets. Data cleansing deals with errors discovered during data entry, practically impossible values, missing values, outliers, spaces, typos, and errors against codebooks. The overall system diagram is shown in Fig. 1.

### Dataset description

A dataset including a wide range of simulated incursions into a military network environment was made available for assessment. By emulating a typical US Air Force LAN, it established a setting for acquiring raw TCP/IP dump data for a network. The LAN was bombarded with several attacks and concentrated like a genuine atmosphere. A connection is a series of TCP packets that begin and stop at specific times and allow data to flow from a source IP address to a target IP address according to a specific protocol. Additionally, each link has a label designating it as either normal or an attack of exactly one particular attack kind. An average connection record is 100 bytes long. 41 quantitative and qualitative features are extracted from normal and attack data for each TCP/IP connection (3 qualitative and 38 quantitative features). Table 2 lists the two categories for the class variable:

**Table 2:** Data classes sample information.

S. no.	Categories	Number of samples
1	Normal	13,449
2	Anomaly	11,743

### Data analysis of network traffic data

This research chooses a robust genetic ensemble classifier random forest model-based bagging model using a genetic algorithm for optimization, as well as Naive Baye, K closest neighbor, decision trees, logistic regression, and bagging as its data analysis strategy. For each approach, models are created and tested using a network traffic dataset. We compared the findings of all models using graphs and performance rates to assess how well they performed in terms of forecasting network incursion. Several procedures are carried out before experiments, including data preparation, data exploration, and model construction^41^. These procedures are crucial for getting data ready so that it may be fed into models. Data transformation involves aggregating data, extrapolating data, creating dummies, and reducing the number of variables. Data cleansing deals with errors discovered during data entry, practically impossible values, missing values, outliers, spaces, typos, and errors against codebooks. Modelling and Prediction deals with all the model configuration, training, testing and optimization process of Robust genetically ensemble model.

### Data analysis of network traffic data logs

Following are the steps we applied for Data Analysis process:

#### Data acquisition

In Data Acquisition, we fetched network traffic train and test data from CSV files and into a data frame.

#### Exploratory data analysis

Exploratory data analysis, also known as EDA, is the process of examining data to identify its key characteristics. This framework was invented by John Tukey, who also wrote a landmark book on the subject (called Exploratory Data Analysis). EDA involves calculating numerical summaries of data, visualizing data in a variety of ways, and considering interesting data points. Before any model fitting is done to the data, some exploratory data analysis should always be performed. EDA appears to be a lot bigger focus in data science than it is in classical statistics. EDA involves computing values and displaying data for the following purposes: Dimension reduction, checking the n's, identifying missing data, characterizing the distributional features of the data, and characterizing relationships between variables and observations, and Model development and hypothesis generating.

##### Data information

In Table 3 data information some details out of 40 different values like data type, null and non-null, class, etc. samples related to data some columns are visualized.

**Table 3:** Data columns information.

	Column	Non-Null	ll Count	Dtype
0	Duration	25,192	non-null	int64
1	protocol_type	25,192	non-null	object
2	Service	25,192	non-null	object
3	Flag	25,192	non-null	object
4	src_bytes	25,192	non-null	int64
5	dst_bytes	25,192	non-null	int64
6	Land	25,192	non-null	int64
7	wrong_fragment	25,192	non-null	int64
8	Urgent	25,192	non-null	int64
9	Hot	25,192	non-null	int64
10	num_failed_logins	25,192	non-null	int64

##### Data description

Table 4 shows data description statistical details like mean, maximum, minimum, 25%, 50%, 75%, counts, and standard deviations samples related to some data columns are visualized.

**Table 4:** Data columns description.

	Count	Mean	Std		
Duration	25,192	305.0541	2686.556		
src_bytes	25,192	24,330.63	2,410,805		
dst_bytes	25,192	3491.847	88,830.72		
Land	25,192	7.94E-05	0.00891		
wrong_fragment	25,192	0.023738	0.260221		
Urgent	25,192	3.97E-05	0.0063		
Hot	25,192	0.198039	2.154202		
num_failed_logins	25,192	0.001191	0.045418		
logged_in	25,192	0.394768	0.488811		
num_compromised	25,192	0.22785	10.41735		
root_shell	25,192	0.001548	0.039316		
su_attempted	25,192	0.00135	0.048785		
num_root	25,192	0.249841	11.50084		
num_file_creations	25,192	0.014727	0.529602		
num_shells	25,192	0.000357	0.018898		
num_access_files	25,192	0.004327	0.098524		

	Min	25%	50%	75%	max
Duration	0	0	0	0	42,862
src_bytes	0	0	44	279	3.82E+08
dst_bytes	0	0	0	530.25	5,151,385
Land	0	0	0	0	1
wrong_fragment	0	0	0	0	3
Urgent	0	0	0	0	1
Hot	0	0	0	0	77
num_failed_logins	0	0	0	0	4
logged_in	0	0	0	1	1
num_compromised	0	0	0	0	884

##### Category to detect

**Figure 2:**
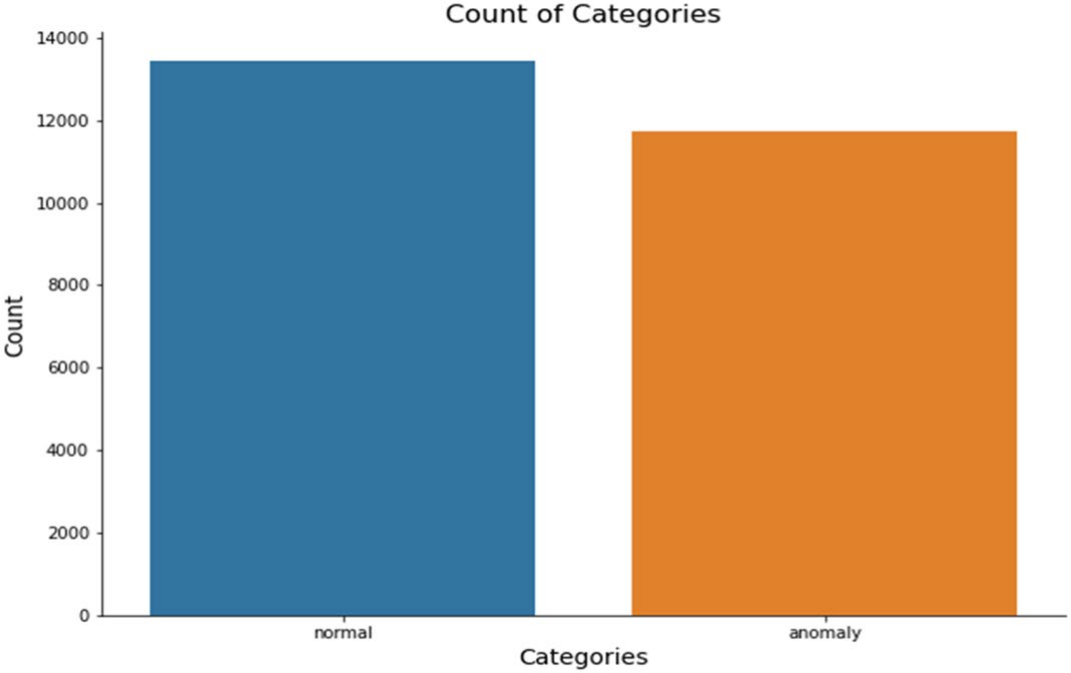
Count Bar plot of intrusion classes.

Class column contain the intrusion categories Normal and Anomaly. In Fig. 2 sample counts of categories are visualized.

##### Qualitative features

Flag and Service columns are the statistics of categorical features, that have a significant impact on intrusion, are broken down below into before and after cleaning statistics. For the study of machine learning models to have the desired input features, these outliers' observations must be deleted from the dataset. Both approaches are quite susceptible to the biases of the outliers.

##### Quantitative features

The quantitative numerical features that have a significant impact on intrusion detection are statistically represented here. According to the presence of outliers and missing data, the skewness vs. density of numerical features. For the study of machine learning models to have the desired input features, these outliers' observations must be deleted from the dataset. Both approaches are quite susceptible to the biases of the outliers.

#### Data preprocessing

**Figure 3:**
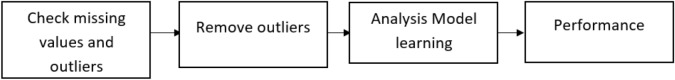
Flow diagram of data pre-processing.

Following are the steps we applied for data preprocessing as shown in Fig. 3.

##### Data cleaning

Data cleaning refers to detecting incomplete, unreliable, inaccurate, or irrelevant aspects of the data and then restoring, rebuilding, or eliminating the filthy or crude data. It also refers to identifying and removing (or correcting) faulty records from a dataset, table, or database. Data cleaning can be done interactively with data-wrangling tools or in batches using scripts. A dataset should be consistent with other associated datasets in the operation after cleaning^42^. The errors made by users when entering data, corruption during storage or transmission, or different data dictionary definitions of identical things in different stores may have been the primary causes of the discrepancies found or eliminated.

##### Removing missing values

Because we have not accurately studied the behavior and interaction with other variables, missing data in the training data set can limit the power/fit of a model or result in a biased model. It could result in incorrect classification or prediction. There are a few explanations for why certain observations in a batch of data are missing. Although such missing numbers are not always incorrect, they are likely to make many analysis techniques more difficult. Outliers and missing data may be linked to excessive levels of noise and disturbance that cause the model's predictions to be incorrect. Before entering data into a model, it is crucial to investigate and identify missing values and outliers because failing to do so could have a serious negative influence on data modeling.

#### Feature selection

To cut down on the number of features, the most important components were picked utilizing a hybrid manual-codes selection technique. To make the analytical procedure simpler, features of type numerical or continuous were chosen out of 40 explanatory features. The choice of these attributes was given top consideration. The unwanted columns were eliminated, but the chosen columns were kept. However, only those characteristics that are important in the machine learning and ensemble learning models will be included in subsequent data reduction from these chosen features. High correlation features are chosen for the initial machine learning and ensemble learning classification models once this correlation matrix for the features is computed.

##### Correlation matrix

Preprocessing the dataset to remove the attributes X1,…. Xp that has the most effects on the target Y is how filter methods operate. The following are a handful of these:

132 Pearson Coefficient This approach makes it simple to filter attributes according to their correlation coefficient. The following Eq. (1) describes the Pearson correlation coefficient between a goal (Y) and a feature (Xi):

1 $$\rho_{i} = \frac{{cov(X_{i} Y)}}{{\sigma (X_{i} )\sigma_{Y} }}$$

where is the standard deviation and cov(Xi,Y) is the covariance^43^. It can be utilized for binary classification and regression problems and ranges from (1,1) from a negative to a positive correlation. It is a brief statistic that ranks the traits according to the target's absolute correlation coefficient Correlation-based feature selection (CFS)^44^. To reduce redundancy and choose a diversified feature set, CFS was developed to choose a subset of features with significant correlation to the aim and low intercorrelation among themselves. In contrast to individual features, the CFS favors a heuristic over a feature subset. The symmetrical uncertainty correlation coefficient is calculated using the formula Eq. (2) below:

2 $$r(X,Y) = 2.0 \times \frac{IG(X|Y)}{{H(X) + H(Y)}}$$

where the sign IG(X|Y) stands for the information provided by feature X for class attribute Y. The variable X has an entropy of H. (X). The following merit metric was used to rank each subset S with k features see Eq. (3):

3 $$Merits = \frac{{k\overline{{r_{cf} }} }}{{\sqrt {k + k(k - 1)\overline{{r_{ff} }} } }}$$

where rff is the average feature-feature inter-correlation and rcf is the mean symmetrical uncertainty correlation between the feature (f S) and the target. CFS is frequently paired with search algorithms like forward selection, backward elimination, and bi-directional search to take into consideration the significant computational burden of evaluating all candidate feature subsets. In this investigation, we used the sci-kit-learn version of CFS^45^, which employs symmetrical uncertainty^46^ as the correlation metric and stops searching the subset space after five successive fully enlarged non-improving subsets.

**Figure 4:**
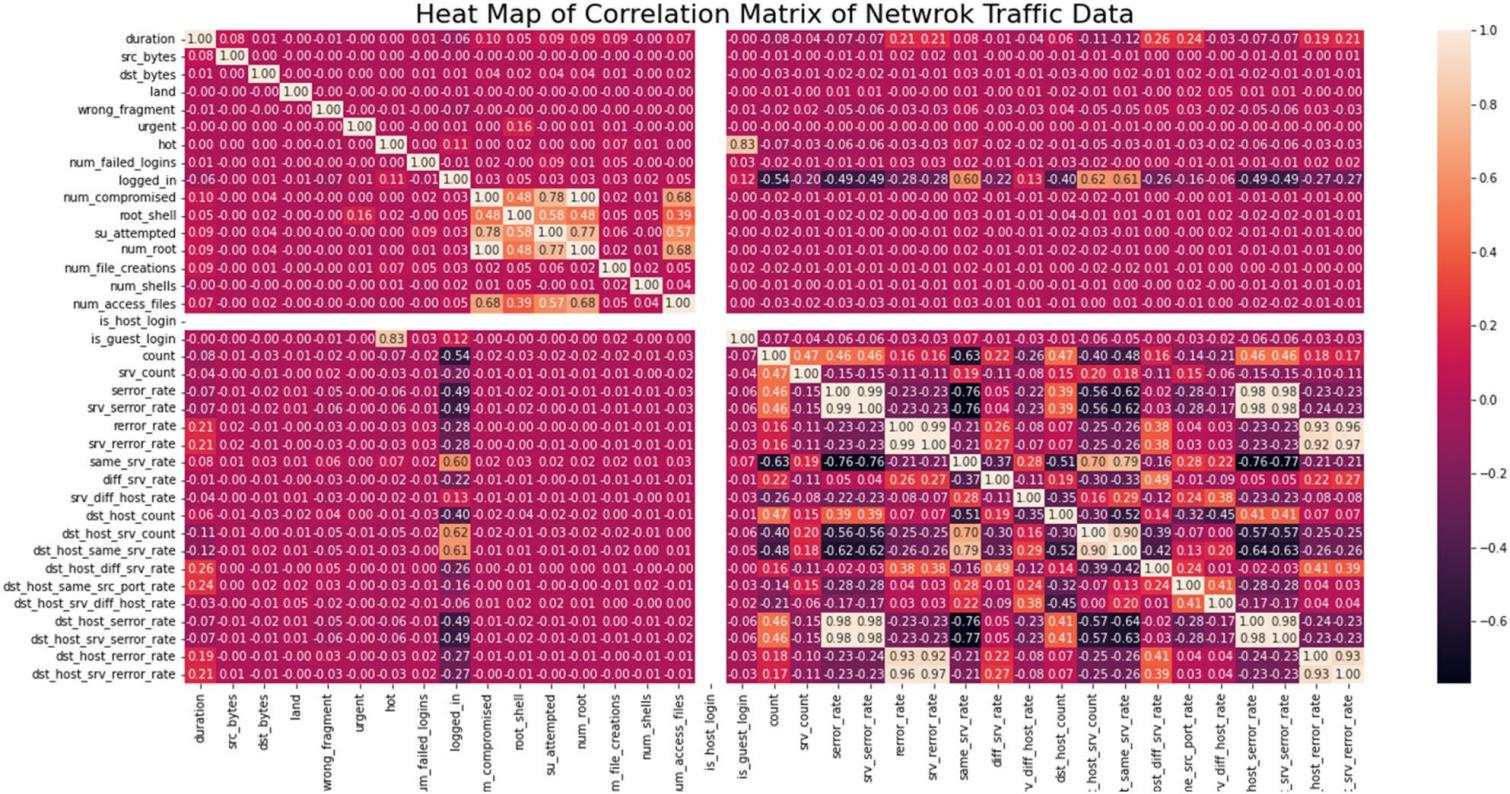
Heat map of network traffic data.

The heat map of the correlation matrix, which displays the most associated features about the class column, is shown in Fig. 4. Table 5 shows the Top most correlated features selected from the correlated list.

**Table 5:** Most correlated features.

	Most correlated features	Score
0	logged_in	0.688084
1	Count	0.57879
2	serror_rate	0.649952
3	srv_serror_rate	0.647817
4	same_srv_rate	0.749237
5	dst_host_srv_count	0.719292
6	dst_host_same_srv_rate	0.692212
7	dst_host_serror_rate	0.65105
8	dst_host_srv_serror_rate	0.653759
9	Class	1

#### Data transformation and feature selection

Data A series of techniques known as feature transformation produce new features (predictor variables). A subset of feature transformation called feature selection is used to find knowledge, improve interpretability, and obtain some understanding, and there are two methods of feature selection due to the curse of dimensionality. Methods of the filter type choose variables without consideration of the model. They are solely dependent on universal characteristics like the correlation with the predicted variable. Filtering techniques eliminate the least intriguing factors. Their primary function is as a pre-processing technique^47^. One more is the evaluation of subsets of variables by wrapper methods, which, in contrast to filter approaches, enables the identification of potential interactions between variables^48^.

**Figure 5:**
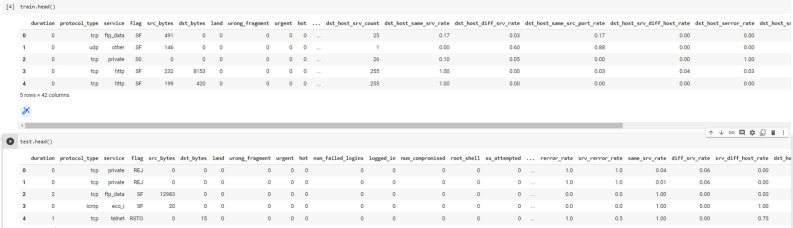
Before feature transformation.

**Figure 6:**
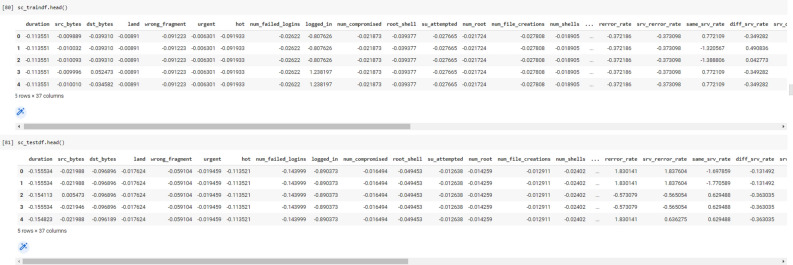
After feature transformation.

In Figs. 5 and 6 show the data before and after transformation like conversion of datatypes and conversion of categorical data into numeric form.

### Machine learning models

These are some fundamental approaches of the used algorithms that need to be understood before moving on to understand the ensemble learning algorithm. Robust genetic ensemble learning algorithms are based on combinations of random forest-based bagging classifier, KNN, decision tree, and logical regression-based voting classifier^49^. To achieve greater predictive performance than a single machine learning classifier, ensemble approaches mix many classifiers. The ensemble model's core tenet is that a collection of weak learners can be combined to create a strong learner, boosting the model's accuracy. Noise, variation, and bias are the key reasons why actual and predicted values differ when we apply any machine learning technique to predict the target variable. Ensemble aids in minimizing these factors (except noise, which is an irreducible error). By using a genetic algorithm, we can increase ensemble learning's accuracy, performance, and computational effectiveness. Aggregating with Bootstrap or bagging^50^.

We looked into how our modeling technique affected the performance of the ensemble using our network traffic dataset. It is important to note that the aim of our research is not to achieve the lowest possible error rate on the datasets, but rather to enhance intrusion detection performance in real-time environments using our Robust genetically Optimized ensemble classifier and to offer a better modeling method for network traffic intrusion detection^51^. Other classification methods that rely on traditional bagging and voting, like decision trees or logistic regression, may be better suitable, especially for straightforward problems. As was previously mentioned, it is difficult to modify these algorithms to transmit preference order as required by the bagging methods employed here^52^.

#### Robust genetically optimized ensemble bagging modelling

Using an evolutionary genetic method, we can optimize the bagging model. GA falls within the broader category of “evolutionary computation,” a soft computing approach. Through a process akin to biological evolution, they aim to find the best answers. To create superior solutions from a pool of current ones, it is necessary to follow the survival of the fittest principle and engage in crossbreeding and mutation. Many different situations for which there are no workable algorithmic solutions are amenable to genetic algorithms' solution-finding abilities. The optimization technique, which seeks one or more extremely good answers from a huge number of potential solutions, is especially well suited to the GA methodology. By continuously ranking the current generation of potential solutions, rejecting the ones considered poor, and creating a new generation by breeding and modifying the ones ranked as good, GA shrinks the search space. Candidate solutions are ranked according to a predetermined standard of goodness or fitness. The implementation of the model is shown in the following algorithm:

**Algorithm 1: Data Analysis Processes**

1. Here data has been collected from train and test data CSV files and organized in a dictionary.
2. Load Data from Train and test Files.
3. Arrange Data in a table form split into columns.
4. Organized data in the Data frame in Table.
5. Give those Columns Names in table form.
6. Combine Train and Test data.
7. Replace the field.
8. Check missing values.
9. Do Statistical Analysis by Plotting counting plots and box graphs of every feature variable and Label.
10. Remove Outliers from Data by dropping columns that are not in valid range of data.
11. Plot Count graphs for statistical analysis after removing outliers.
12. Check the most correlated matrix of features find the top 10 most correlated features plot heatmaps and list down the list of those features’ names.
13. Drop unwanted columns from the data table.
14. Save the transformed dataset for Modelling and prediction.
15. Split data for training and testing with a ratio 0.3 for testing and validation.0.7 for Training.

#### Naïve Bayes

A Naive Bayes classifier is a straightforward probabilistic classifier built using the Bayes theorem and naive independence assumptions from Bayesian statistics. The phrase “independent feature model” might be a better way to describe the underlying probability model. A naïve Bayes classifier, to put it simply, thinks that the presence (or lack) of one feature in a class has nothing to do with the presence (or absence) of any other feature. In a supervised learning environment, naive Bayes classifiers can be trained quite effectively depending on the specifics of the probability model^53^. It is possible to operate with the naive Bayes model without accepting Bayesian probability or applying any Bayesian techniques because parameter estimation for naive Bayes models frequently employs the maximum likelihood method, as shown in Eq. (4)

4 $${\text{posterior}} = \frac{{{\text{prior}} \times {\text{likelihood}}}}{{{\text{evidence}}}}$$

#### K nearest neighbor (KNN)

KNN, also known as K Nearest Neighbors, is currently one of the most well-known classification algorithms in the industry because it is so simple and reliable. KNN is a simple algorithm that records all real-time cases and categorizes new cases based on a similarity metric. Since the 1970s, statistical estimation and pattern recognition have used KNN as a non-parametric method. The technique classifies values that are close together and have similar values. In the KNN model, K is the number of nearest neighbors. The number of neighbors is the main determining factor. In cases when there are two classes, K is often set to be odd. When K = 1, the basic example describes the process as the nearest neighbor algorithm. Euclidean distance, commonly referred to as simply distance, is the most widely used unit of measurement for distance. A Euclidean distance metric should be used when working with dense or continuous data, according to many research. Euclidean distance is the most accurate way to determine proximity. The path's length is known as the Euclidean distance between two locations, as shown in Eq. (5)

5 $${\mathrm{Euclidean\, distance\, formula}}: \sqrt{\sum_{i=1}^{k}({x}_{i}-{y}_{i})}$$

#### Decision tree

The supervised learning algorithms family includes the decision tree algorithm. The decision tree technique, in contrast to other supervised learning methods, is capable of handling both classification and regression issues. By learning straightforward decision rules derived from previous data, a Decision Tree is used to build a training model that may be used to predict the class or value of the target variable (training data). In decision trees, we begin at the tree's root when anticipating a record's class label. We contrast the root attribute's values with that of the attribute on the record. We follow the branch that corresponds to that value and go on to the next node based on the comparison.

#### Logistic regression

One of the most often used Machine Learning algorithms, within the category of Supervised Learning, is logistic regression. Using a predetermined set of independent factors, it is used to predict the categorical dependent variable. In a categorical dependent variable, the output is predicted via logistic regression. As a result, the result must be a discrete or categorical value. Rather than providing the exact values of 0 and 1, it provides the probabilistic values that fall between 0 and 1. It can be either Yes or No, 0 or 1, true or false, etc. Except how they are applied, logistic regression and linear regression are very similar. While logistic regression is used to solve classification problems, linear regression is used to solve regression problems. The Logistic regression equation can be obtained from the Linear Regression equation. The mathematical steps to get Logistic Regression equations are given below in Eq. (6):

6 $$y = b_{0} + b_{1} x_{1} + b_{2} x_{2} + b_{3} x_{3} + \cdots + b_{n} x_{n}$$

#### Bagging classifier

**Figure 7:**
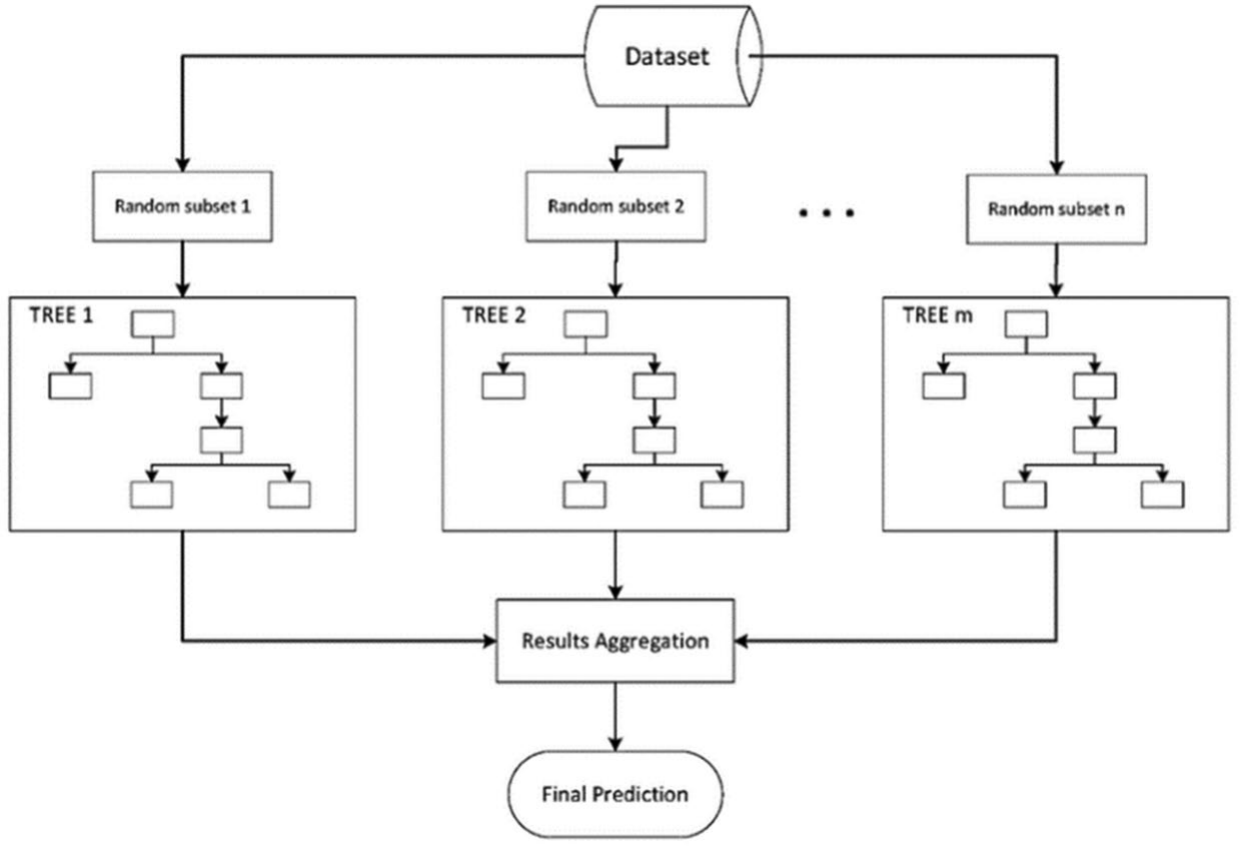
Bagging decision process.

Because it combines Bootstrapping and Aggregation to create a single ensemble model, Bagging gets its name. Multiple bootstrapped subsamples are taken from a sample of data. On each of the bootstrapped subsamples, a Decision Tree is constructed. An algorithm is used to aggregate across the Decision Trees to create the best effective predictor once each subsample Decision Tree has been created. The illustration in Fig. 7.

Bootstrapped subsamples are drawn from a dataset. On each bootstrapped sample, a Decision Tree is created. The strongest, most precise predictor is produced by averaging the outcomes of each tree. The concept of bagging can be used for random forest models with a minor modification. Bagged Decision Trees have a complete range of characteristics at their disposal to pick from when deciding where to split and how to make decisions. As a result, even if the bootstrapped samples may differ slightly, the data will typically split off at the same characteristics for each model. On the other hand, Random Forest models select features at random to determine where to split. Because each tree will split based on different features, Random Forest models incorporate a level of differentiation as opposed to splitting at similar features at each node throughout.

## Result and discussions

### Performance evaluation matrices

As a result, using the actual and forecast array values, we may assess the Mean Absolute Error (MAE), Mean Square Error (MSE), and Root Square Score/Variance. These metrics or comparison criteria are defined by the following mathematical formula (7):

7 $$MAE=\frac{1}{n}\sum_{i=1}^{n}|({y}_{i}- \widehat{{y}_{i}})|$$

where $n$ is the number of observations, ${y}_{i}$ is the values of the observations and $\widehat{{y}_{i}}$ is the predicted values in (8)

8 $$MSE=\frac{1}{n}{\sum_{i=1}^{n}({y}_{i}- \widehat{{y}_{i}})}^{2}$$

where $n$ is the number of observations, ${y}_{i}$ is the values of the observations as

9 $${R}^{2}=1- \frac{\sum_{i=1}^{n}({y}_{i}- \overline{y })({y}_{i}-\overline{\widehat{{y }_{i}}} )}{\sum_{i=1}^{n}({y}_{i}- \overline{y })}$$

The mean of the actual value is, the mean of the predicted value is $\overline{y }$ and $n$ is the number of observations, where ${y}_{i}$ I represent the actual value and $\overline{y }$ represents the anticipated value. Performance for categorization issues at various threshold levels is measured by the AUC-ROC curve. AUC stands for the level or measurement of separability, and ROC is a probability curve. It illustrates how well the model is in differentiating between classes. The model performs better at classifying the 0 class as 0, and the 1 class as 1, the higher the AUC. The model performs better at differentiating between patients who have the condition and those who do not as the AUC increases.

Other performance evaluation matrices are Recall, Specificity, and FPR as shown in Eq. 10,11,12

10 $$RECALL= \frac{TP}{TP+FN}$$

11 $$SPECIFICITY=\frac{TN}{TN+FP}$$

12 $${\text{FPR}} = 1 - {\text{SPECIFICITY}}$$

### Robust genetic algorithm optimized ensemble model results

**Figure 8:**
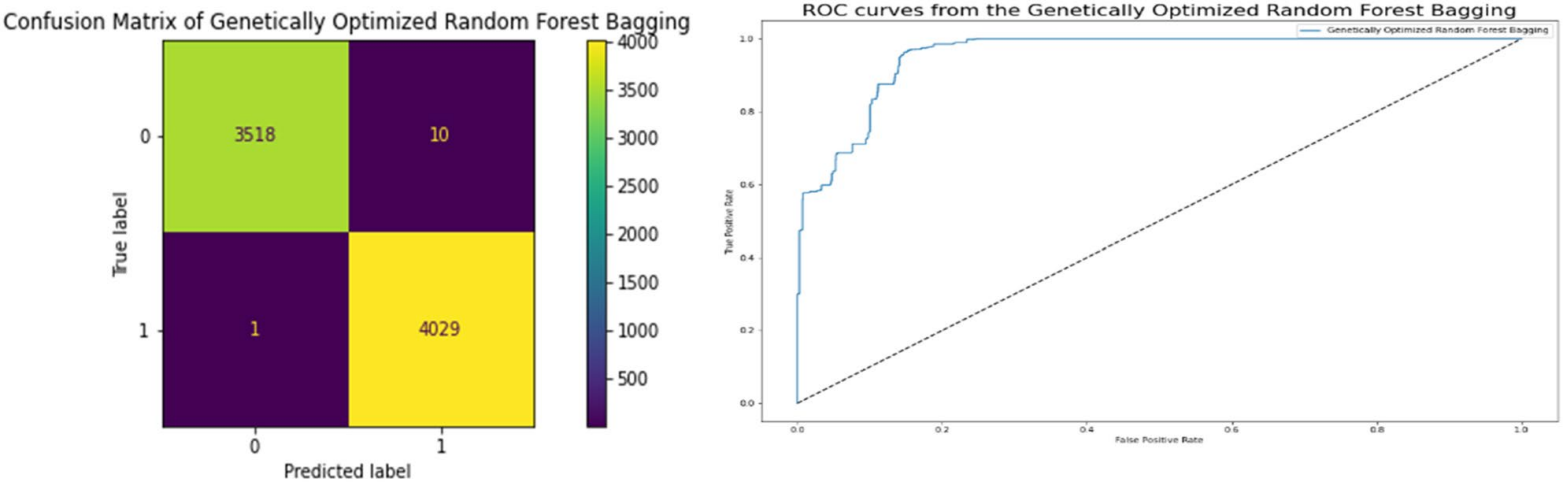
Confusion matrices and ROC curve between targeted and predicted test dataset by robust genetic algorithm optimized ensemble model with selected features.

The performance of the Robust Genetic Algorithm Optimized Bagging Model, which is highly correlated to a better model, is displayed in Table 6. Accuracy in terms of the correlation between the actual and predicted variables is 0.9985, and they achieve a 0.9965 Cross-Validation Score, which is a very strong correlation more than other classifiers. Figure 8 displays the network intrusion Robust Genetic Algorithm Optimized Bagging Model. Using the model's ROC curve and confusion matrix, we can see that the performance demonstrates superior classification. The high AUC on the ROC curve indicates lower mistake rates and higher accuracy. The plot of the model's predicted result against the actual words states how well the model fits the data. Root Square Score is 0.9941, which is fairly high compared to other classifiers, and the Mean Absolute Error and Mean Square Error are both 0.00145, indicating that the model performed well on the Network Traffic Dataset.

**Table 6:** Performance robust genetic algorithm optimized ensemble model results.

Model	Genetically optimized random forest bagging
Cross Validation Score	0.996528
Accuracy	0.998545
Mean Square Error	0.001455
Mean Absolute Error	0.001455
Root Square Score/Variance	0.994153

### Naïve Baye classifier results

**Figure 9:**
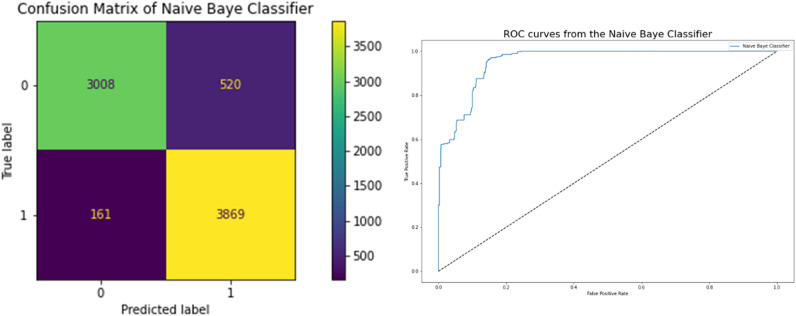
Confusion matrices and ROC between targeted and predicted test dataset without noise by Naïve Baye Classifier.

In Table 7, the effectiveness of the Naive Baye Classifier, which has a strong correlation with better models, is displayed. Its accuracy is 0.909 in terms of the correlation between the predicted and actual variables, and it achieves a 0.909 Cross Validation Score. Figure 9 displays the network intrusion Naive Baye Classifier. By examining the model's ROC curve and confusion matrix, we can see that the performance graphs in Fig. 9 demonstrate improved classification. A low AUC on the ROC curve indicates greater accuracy and lower error rates. Root Square Score is 0.63, which is average, and the Mean Absolute Error and Mean Square Error are both 0.0901, indicating that the model did well on the Network Traffic Dataset.

**Table 7:** Performance Naïve Baye classifier results.

Model	Naive Baye classifier
Cross Validation Score	0.909238
Accuracy	0.909897
Mean Square Error	0.090103
Mean Absolute Error	0.090103
Root Square Score/Variance	0.63799

### Decision tree classifier results

**Figure 10:**
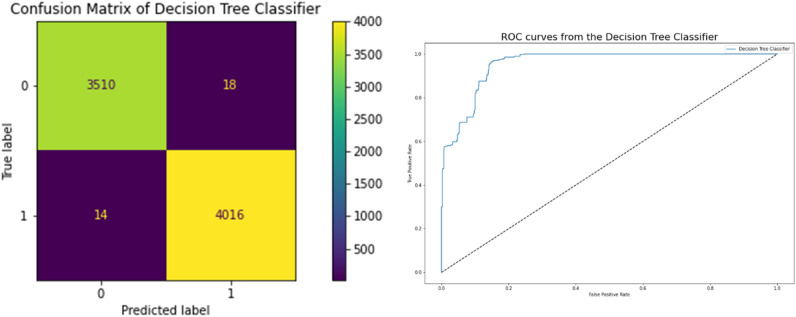
Confusion matrices between targeted and predicted test dataset without noise by Decision Tree Classifier.

The performance of the decision tree classifier, which is highly associated with a better model, is displayed in Table 8. Accuracy is 0.995 in terms of the correlation between the actual and predicted variables, and they achieve a 0.993 Cross-Validation Score, which is a very strong correlation. Figure 10 displays the network intrusion Decision Tree Classifier. Using the model's ROC curve and confusion matrix, we can see that the performance graphs in Fig. 10 demonstrate superior classification. The high AUC on the ROC curve indicates lower mistake rates and higher accuracy. It is believed to show how well the model fits the data. and 0.004 Mean Absolute Error, Mean Square Error, and a High Root Square Score of 0.98, demonstrating the model's success on the Network Traffic Dataset.

**Table 8:** Performance decision tree results.

Model	Decision tree classifier
Cross Validation Score	0.993253
Accuracy	0.995766
Mean Square Error	0.004234
Mean Absolute Error	0.004234
Root Square Score/Variance	0.982989

### K nearest neighbor

**Figure 11:**
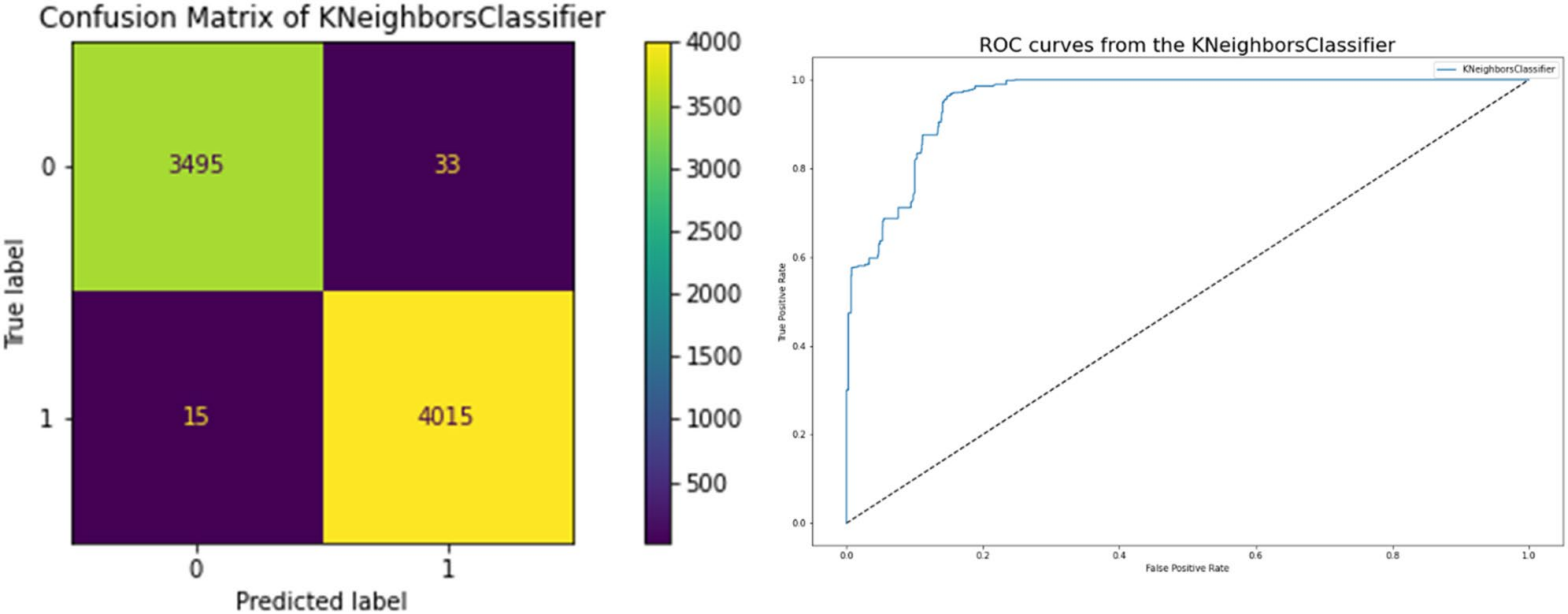
Confusion matrices between targeted and predicted test dataset without noise by K Nearest Neighbors Model.

The performance of the K Nearest Neighbors Classifier, which is highly associated with a better model, is displayed in Table 9. Accuracy in terms of the correlation between the actual and predicted variables is 0.993, and they achieve a 0.988 Cross-Validation Score, which is a very strong correlation. Figure 10 displays the network incorporating the K Nearest Neighbor Classifier. Using the model's ROC curve and confusion matrix, we can see that the performance graphs in Fig. 11 demonstrate superior classification. The high AUC on the ROC curve indicates lower mistake rates and higher accuracy. Root Square Score is 0.97, which is fairly high, and the Mean Absolute Error and Mean Square Error are also 0.006, indicating that the model did well on the Network Traffic Dataset.

**Table 9:** Performance K nearest neighbors model results.

Model	K neighbors classifier
Cross Validation Score	0.988753
Accuracy	0.993649
Mean Square Error	0.006351
Mean Absolute Error	0.006351
Root Square Score/Variance	0.974484

### Logistic regression classifier results

**Figure 12:**
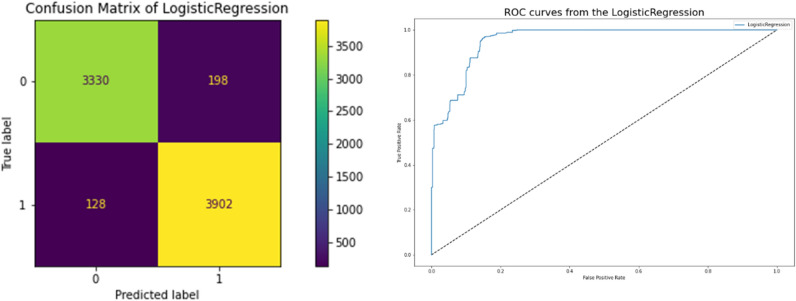
Confusion matrices between targeted and predicted test dataset without noise by Logistic Regression Classifier.

The accuracy of the Logistic Regression Classifier is 0.958 in terms of the correlation between the actual and predicted variables, and they achieve a 0.956 Cross-Validation Score, which is a strong correlation but not better than the Decision Tree. Table 10 presents the performance of the classifier, which is highly correlated to a better model. Figure 12 displays the network intrusion Logistic Regression Classifier. Using the model's ROC curve and confusion matrix, we can see that the performance graphs in Fig. 12 demonstrate superior classification. The high AUC on the ROC curve indicates lower mistake rates and higher accuracy, this plot is believed to show how well the model fits the data. Root Square Score is 0.82, which is fairly high, and Mean Absolute Error and Mean Square Error are both 0.043, indicating that the model did well on the Network Traffic Dataset.

**Table 10:** Performance logistic regression classifier results.

Model	Logistic regression
Cross Validation Score	0.958192
Accuracy	0.956867
Mean Square Error	0.043133
Mean Absolute Error	0.043133
Root Square Score/Variance	0.826703

### Bagging classifier

**Figure 13:**
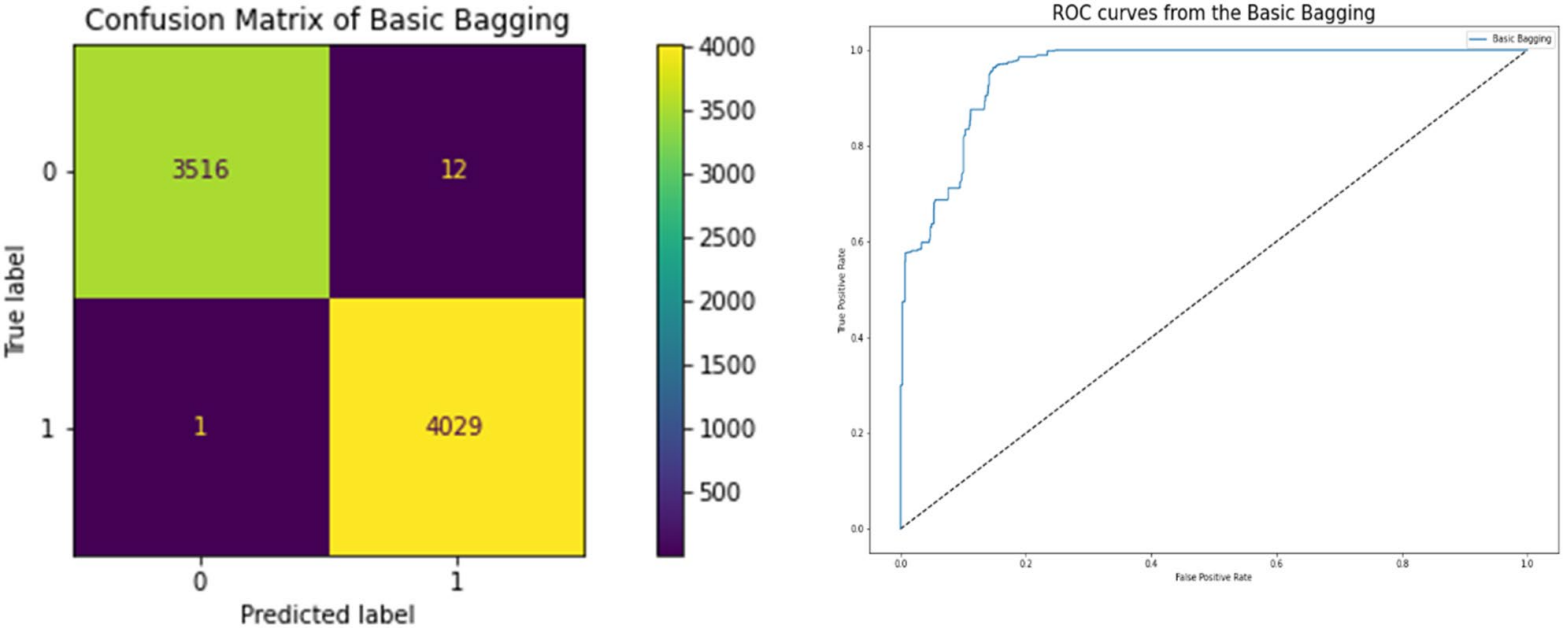
Confusion matrices between targeted and predicted test dataset without noise by Bagging Classifier.

The performance of the Bagging Classifier, which is highly correlated to a better model, is displayed in Table 11. Accuracy in terms of the correlation between the actual and predicted variables is 0.9982, and they achieve a 0.9964 Cross-Validation Score, which is a very strong correlation more than other classifiers. Figure 13 displays the network intrusion Bagging Classifier. Using the model's ROC curve and confusion matrix, we can see that the performance graphs demonstrate superior classification. The high AUC on the ROC curve indicates lower mistake rates and higher accuracy, Root Square Score is 0.9930, which is fairly high compared to other classifiers, while Mean Absolute Error and Mean Square Error are both 0.00172, indicating that the model performed well on the Network Traffic Dataset.

**Table 11:** Performance bagging classifier results.

Model	Basic bagging
Cross Validation Score	0.996428
Accuracy	0.99828
Mean Square Error	0.00172
Mean Absolute Error	0.00172
Root Square Score/Variance	0.993089

### Comparison of the classification accuracy of robust ensemble model for distant and noisy data recognition with traditional machine learning classifications models

**Figure 14:**
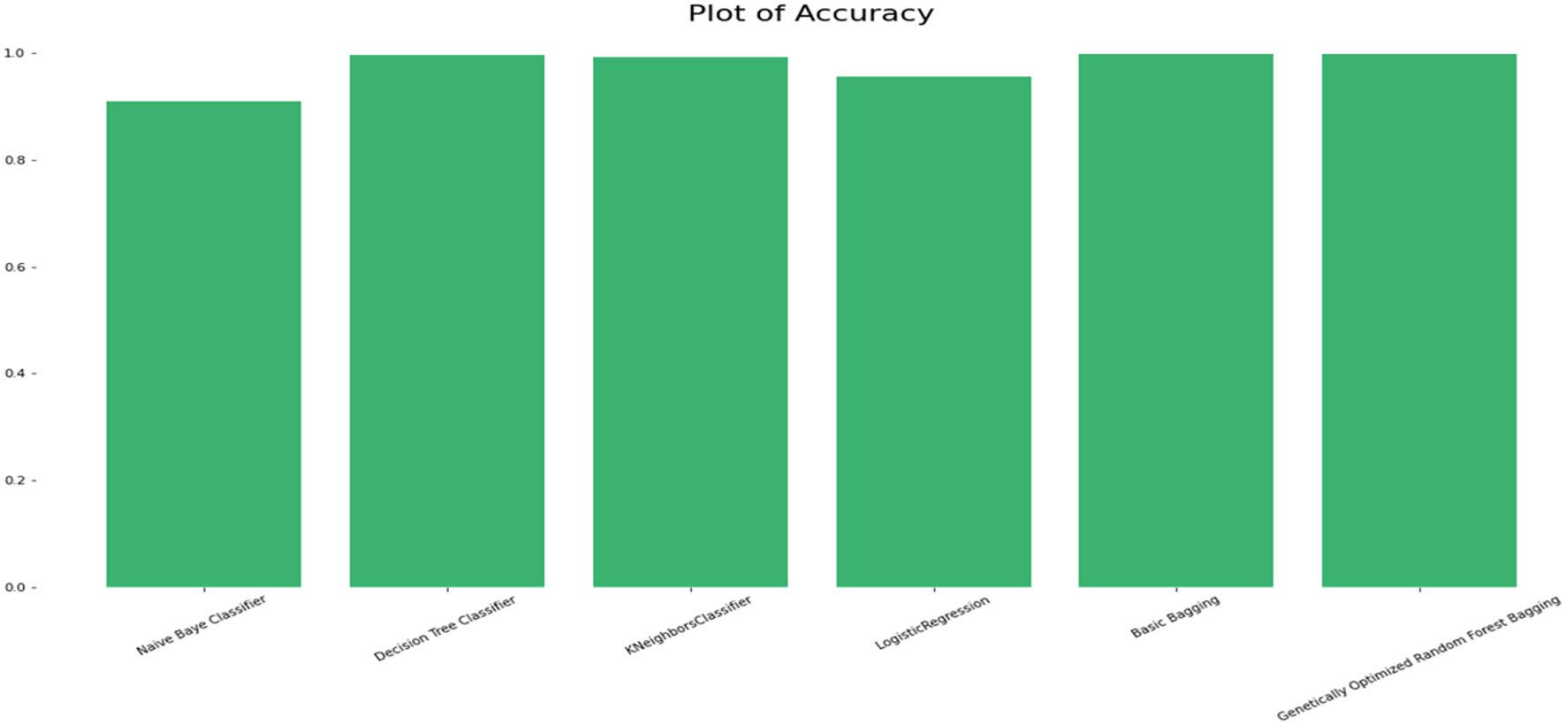
Accuracy comparison plot of machine learning and ensemble learning models with robust genetic algorithm optimized ensemble model.

**Figure 15:**
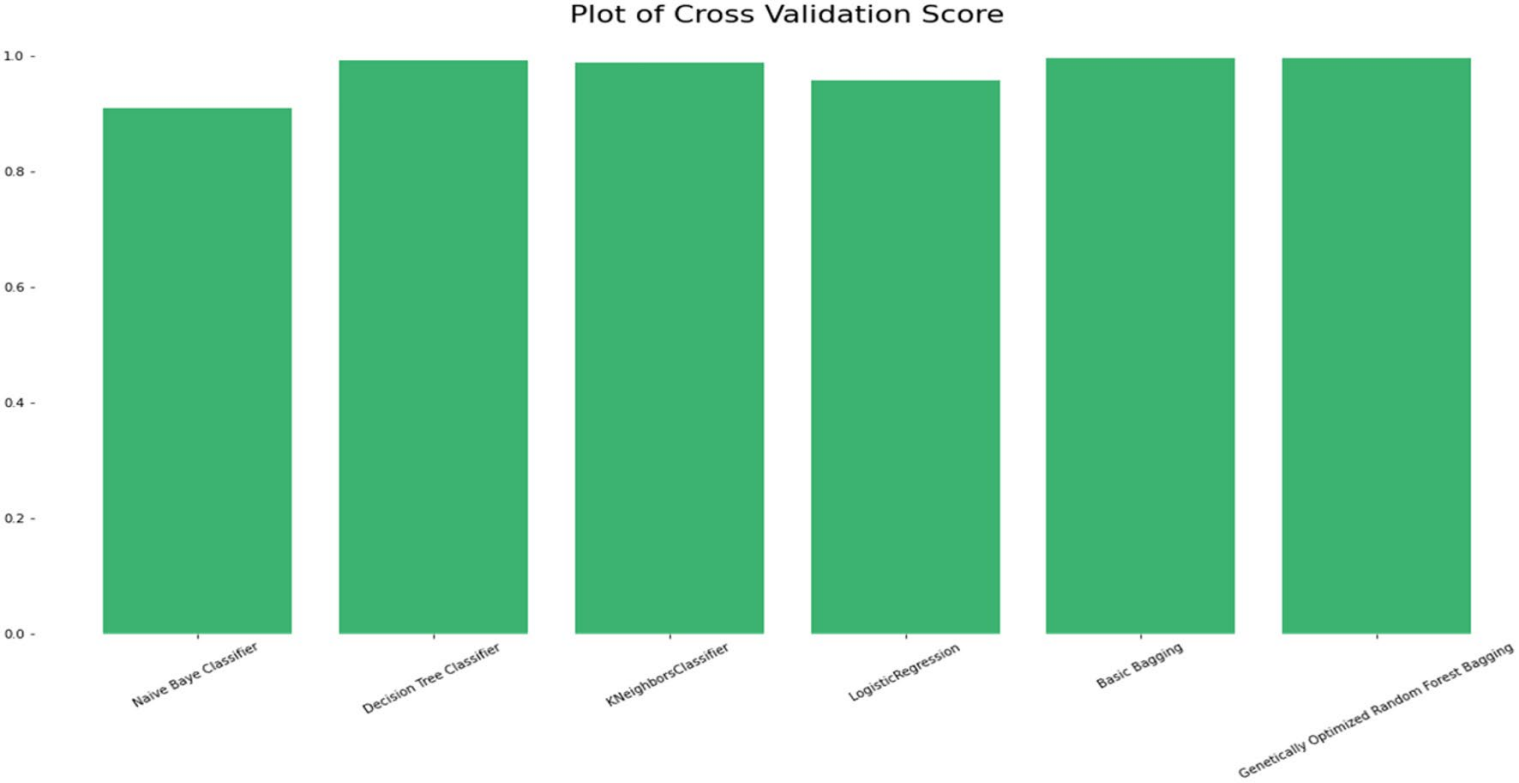
Cross validation score accuracy comparison plot of machine learning and ensemble learning models with robust genetic algorithm optimized ensemble model.

The performance comparison between a Robust Genetic Algorithm Optimized Ensemble Model and other simple models, including the number of errors the ensemble committed over the entire dataset, is shown in Tables 12 and 13. The effectiveness of the simple classifiers (i.e., the traditional variant of each single classifier) is also supplied as a comparison tool for fold cross-validation and on the training set alone. Information for the Six-comparison criterion of models in circumstances without is provided in Tables 12 and 13 and Figs. 14 and 15. This criterion compares the effectiveness of all algorithms with the performance of the chosen features.

**Table 12:** Comparison criterion of models.

Model	Cross validation score	Accuracy	Root square score/variance
Naive Baye Classifier	0.909238235	0.909896798	0.637990159
Decision Tree Classifier	0.993252917	0.995766076	0.982989259
K Neighbors Classifier	0.988753285	0.993649114	0.974483888
Logistic Regression	0.958192298	0.956866896	0.826703072
Basic Bagging	0.996427695	0.998279968	0.993089386
Genetically Optimized Random Forest Bagging	0.99642752	0.998544589	0.994152558

**Table 13:** Comparison criterion of models.

Model	Mean square error	Mean absolute error
Naive Baye Classifier	0.090103202	0.090103202
Decision Tree Classifier	0.004233924	0.004233924
K Neighbors Classifier	0.006350886	0.006350886
Logistic Regression	0.043133104	0.043133104
Basic Bagging	0.001720032	0.001720032
Genetically Optimized Random Forest Bagging	0.001455411	0.001455411

**Figure 16:**
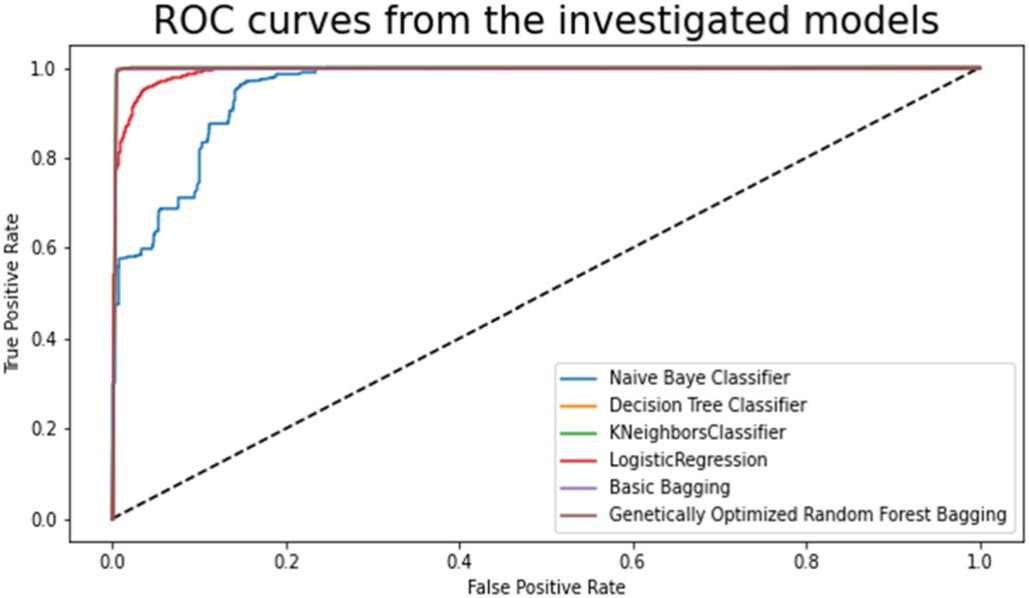
ROC comparison plot of machine learning and ensemble learning models with robust genetic algorithm optimized ensemble model.

**Figure 17:**
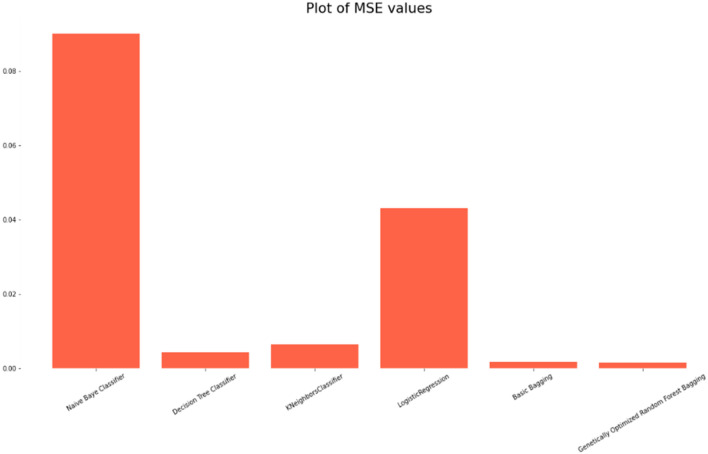
MSE Comparison plot of machine learning and ensemble learning models with robust genetic algorithm optimized ensemble model.

**Figure 18:**
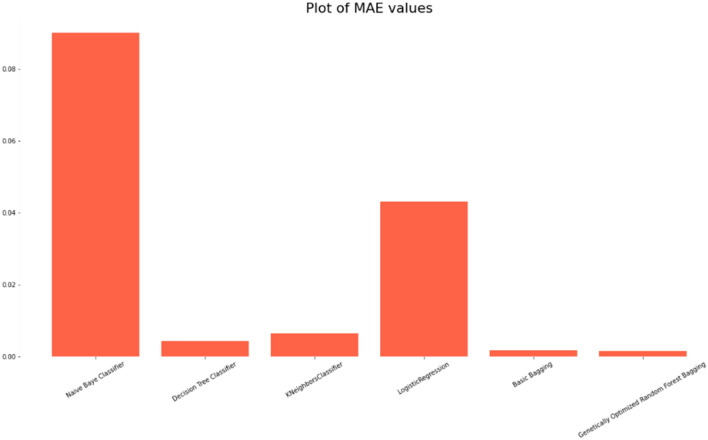
MAE comparison plot of machine learning and ensemble learning models with robust genetic algorithm optimized ensemble model.

**Figure 19:**
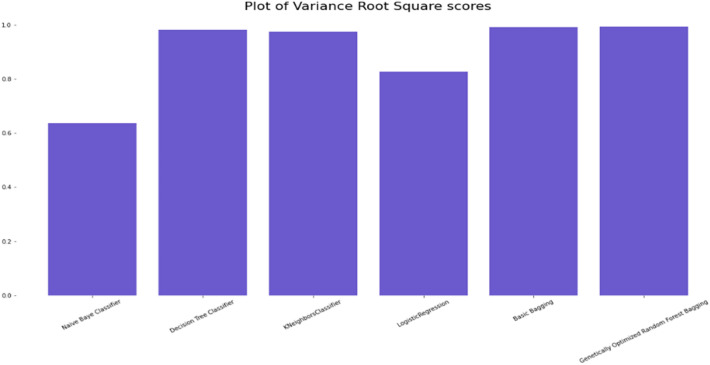
Variance comparison plot of machine learning and ensemble learning models with robust genetic algorithm optimized ensemble model.

The Selected Features with Robust Genetic Algorithm Optimized Ensemble Model performs better than other machine learning models and ensemble learning as shown in Fig. 16, comparison of the ROC curve the AUC is so low as and as it is lower in MAE & MSE, representing the lower the error the better a model and it has lower error rate in comparison of other models as shown in Table 7 and Figs. 17, 18. As shown in Table 8 and Fig. 19, it is also greater in Accuracy Score, variance/Root Square Score, and Cross-Validation Accuracy Score, which indicate that the better a model, the higher the correlation. Due to its superior performance in all two metrics, the Robust Genetic Algorithm Optimized Ensemble Model surpasses the other models.

The Selected Feature with Robust Genetic Algorithm Optimized Ensemble Model performed highly correlated to a better model, which are 0.9985 accuracies with respect to the correlation between actual and predicted variable, 0.9964, which are very strong correlation with the best-fitted model. All algorithms perform reasonably well in terms of the goodness of fit of the model as indicated by the Score.

### Comparison with state of art model

**Table:** 

	Problem	Algorithm used	Results	Defects
Designing a Network Intrusion Detection System Based on Machine Learning for Software Defined Networks^38^	Intrusion detection system based on ML	Decision Tree, Random, Forest, and XGBoost	95% Accuracy	Fewer features used in the predictions of attacks
Proposed Methodology	Intrusion detection system based on Robust Genetic algorithm	Voting Classifier, Bagging Classifier, Gradient Boosting Classifier, and Random Forest-based Bagging algorithm along with the proposed Robust genetic ensemble classifier	99% Accuracy	

## Conclusion

In the pursuit of network security, intrusion detection systems (IDS) play a critical role by meticulously scrutinizing network traffic to ensure the preservation of confidentiality, integrity, and availability. However, despite the dedication of researchers, IDS continues to grapple with challenges in reducing false alarm rates, enhancing detection accuracy, and identifying new intrusion patterns. We proceed to outline the feature selection and analysis procedure, encompassing data gathering, preprocessing, and the selection of influential attributes. The chosen features, including src_bytes, srv_count, dst_bytes, and others, collectively bolster the model's performance. Our results underscore the efficacy of the selected features, particularly in conjunction with ensemble models, leading to notable improvements. Of significance is the pronounced success of the Robust Genetic Algorithm Optimized Ensemble Model, exhibiting remarkable accuracy with a correlation coefficient of 0.9985 between actual and predicted variables, and a strong correlation coefficient of 0.9964—a testament to its model fitting prowess. All algorithms demonstrate commendable performance, as evidenced by the goodness of fit, as indicated by the Score metric.

Looking ahead, the proposed method holds promise for future extensions. The integration of additional machine learning ensemble classifiers and deep learning techniques, coupled with algorithmic enhancements, could yield further advancements on larger datasets. To enhance the practical applicability, future research could explore the incorporation of real-time data streaming and dynamic adaptation mechanisms, enabling the intrusion detection system to promptly respond to evolving threats. Furthermore, collaborations between cybersecurity experts and domain-specific practitioners can lead to refined models that cater to distinct industry nuances.

## Data Availability

All data generated or analyzed during this study are included in this published article.
